# Evidence for glycosylation as a regulator of the pigmentary system: key roles of sialyl(α2-6)gal/GalNAc-terminated glycans in melanin synthesis and transfer

**DOI:** 10.1007/s10719-015-9605-7

**Published:** 2015-07-22

**Authors:** Ganesh Diwakar, Vincent Klump, Rossitza Lazova, John Pawelek

**Affiliations:** Analytical Sciences, The Amway Corporation, Ada, MI USA; Department of Dermatology, Yale School of Medicine, New Haven, CT USA; Department of Dermatology and the Yale Cancer Center, Yale School of Medicine, 333 Cedar Street, PO Box 208059, New Haven, CT 06520-8059 USA

**Keywords:** Sialyl conjugates, Melanocyte, Keratinocyte, Melanin transfer, Skin pigmentation

## Abstract

The major regulators of melanogenesis are glycoproteins, however no role for glycosylation in the pathway has yet been described. We stained skin biopsies and melanocyte-keratinocyte co-cultures with a panel of 20 lectins as oligosaccharide markers. Notably, the Elderberry Bark Lectin (EBL/SNA) stained melanocytes in both systems. EBL binds the sequence Neu5Ac(α(2-6)Gal/GalNAc)- at the termini of some oligosaccharide antennae. We used inhibitors of synthesis and/or binding of this sequence to assess effects on pigmentation. METHODS. Cell culture, lectin histochemistry, siRNA transfection, and assays for dopa oxidase and melanin were carried out by standard techniques. RESULTS. 6′-sialyllactose, a short homolog of the sequence in question, anti-sialyltransferase 6 (ST6) siRNA, and cytidine, a sialyltransferase (ST) inhibitor, each inhibited EBL binding, melanogenesis and melanosome transfer. Unexpectedly, 3′-sialyllactose and siRNA for ST3, chosen as a negative controls, also inhibited these processes. Though strong inhibitors of melanization, none of the agents affected tyrosinase/dopa oxidase activity, indicating previously unrecognized post-tyrosinase regulation of melanization. CONCLUSIONS. We report for the first time that Neu5Ac (α(2-6)Gal/GalNAc)- and possibly Neu5Ac(α(2–3)Gal/GalNAc)-terminated oligosaccharides play multiple roles in melanin synthesis and transfer.

## Introduction

Melanosome transfer from melanocytes to keratinocytes is a unique biological process involving organelle donation from one cell to another and a crucial step in skin pigmentation. Individuals with defects in transfer can have markedly reduced skin melanin content [1–3]. Melanosome transfer begins with attachment of melanocyte dendrites to keratinocytes followed by transfer of melanosomes through the melanocyte dendrites into the keratinocytes and finally trafficking of the melanin within keratinocytes to the supra-nuclear area of the cell. There is growing information on melanocyte-keratinocyte transfer regarding cell biology, cytokine and hormonal signaling pathways and the role of various peptides and proteins [4–8]. However, while the major protein regulators are glycoproteins, little is known about the roles of glycosylation in the process. Accordingly, we assembled a panel of 20 biotinylated lectins as markers for specific glycosylation structures and used lectin histochemistry to analyze staining patterns in biopsies of human skin and co-cultures of human melanocytes and keratinocytes. The Elderberry Bark lectin (EBL) showed specific staining of melanocytes and highlighted melanocyte dendrites. Here we report on the EBL binding site and its roles in melanin synthesis and transfer.

## Experimental results

### Lectin binding studies in cutaneous biopsies

While most of the 20 lectins studied showed no specific staining of melanocytes, EBL was notable because it strongly stained melanocytes compared to other cells in the epidermis, with prominent labeling of dendrites. EBL recognizes the terminal Neu5Ac(α(2-6)Gal/GalNAc)- sequence on certain glycans. The same staining patterns were seen in biopsies from individuals of a variety of ethnic backgrounds and skin colors (not shown).

**Fig. 1:**
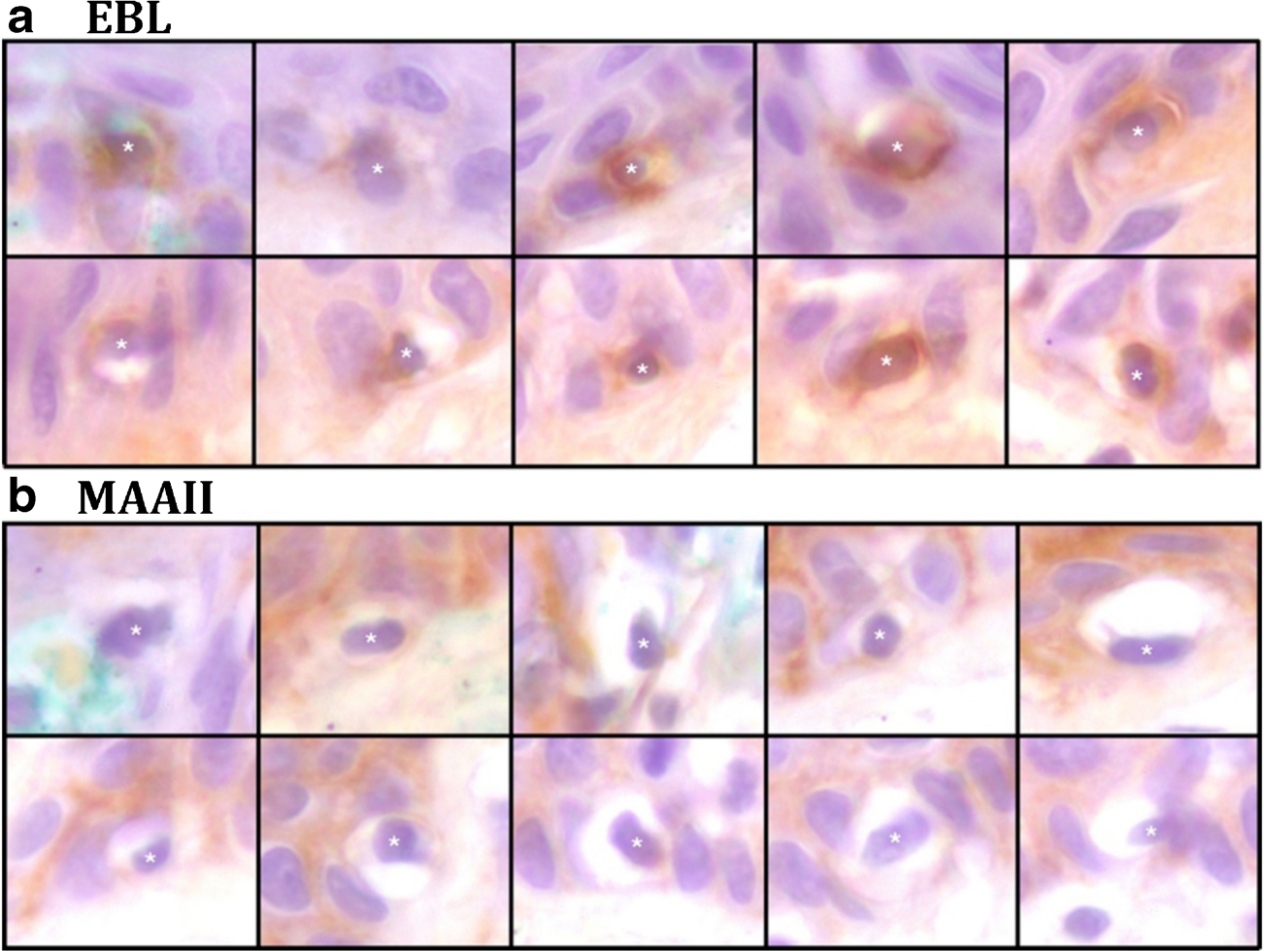
Histochemical staining of melanocytes from sequential sections of the same skin biopsy. Sections were stained through standard immunoperoxidase methods using a *brown* chromagen. Counter-staining was with hematoxylin (*blue*). **a**. A section stained with EBL lectin showing *brown* staining of melanocytes. **b**. A sequential section stained with MAAII lectin. Melanocyte nuclei are marked with *white asterisks*

Histological sections of skin biopsies were stained with EBL and as a control the MAAII lectin (*Maackia amurensis* L.) that had shown little or no staining of melanocytes in the 20-lectin survey above. MAAII recognizes the Neu5Ac(α(2-3)Gal/GalNAc)- sequence. While EBL again stained melanocyte dendrites emanating from the cell body (Fig. 1a), the MAAII lectin did not (Fig. 1b). These findings demonstrated the specificity of the EBL lectin and the Neu5Ac(α2-6)Gal/GalNAc- sequence for melanocytes.

### EBL binding in melanocyte-keratinocyte co-cultures

**Fig. 2:**
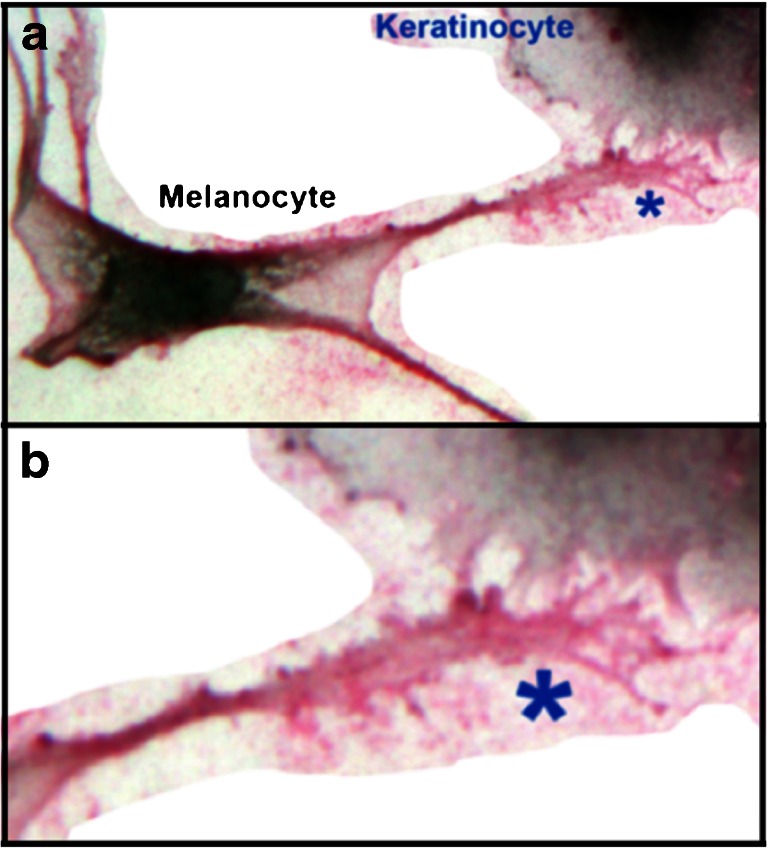
A photograph of a melanocyte in contact with a keratinocyte in co-culture. Cultures were stained with EBL by standard histochemical techniques using a red chromagen and photographed through a Zeiss light microscope (see “Materials and Methods”). **a**. Low power photo showing EBL staining of melanocyte plasma membrane. **b**. High power of the filapodial contact points (*blue asterisk*)

We next investigated EBL staining in co-cultures of human melanocytes and keratinocytes, in this case with a red chromagen. Melanocytes were identified in culture by their dark pigmentation and elongated dendrites. Keratinocytes were non-pigmented with punctate dendrites. Figure 2a shows a melanocyte in contact with a keratinocyte. The melanocyte plasma membrane, including dendrites, stains strongly with EBL. At points of contact with the keratinocyte, the melanocyte dendrite extends numerous filapodia that also stain with EBL (blue asterisk). A higher power view is shown in Fig. 2b. This close association indicated that oligosaccharides terminated with Neu5Ac(α(2-6)Gal/GalNAc)- are likely to function in melanosome transfer. Interestingly, Neu5Ac(α(2-6)Gal/GalNAc)- is the terminal sequence for some membrane-associated glycoconjugates in various biological systems [9]. Synthesis of this sequence is catalyzed by β-galactoside (α2,6)-Sialyltransferase 6 (ST6Gal.I) that catalyzes formation of Neu5Ac(α2-6)Gal/GalNAc- terminus of some N- and O-linked oligosaccharides. Binding is highly specific, as the EBL discriminates between the Neu5Ac(α(2–6)Gal/GalNAc)- sequence and the related Neu5Ac(α2-3)Gal/GalNac- sequence recognized by MAAII due to steric hindrance [10, 11]. These results demonstrate that the Neu5Ac(α(2-6)Gal/GalNAc)- sequence is at the terminus of glycans on melanocyte dendrites, where it is likely to be involved with melanosome transfer to keratinocytes.

Involvement of Neu5Ac(α(2-6)Gal/GalNAc)- linkages and ST6Gal.I represent a previously unrecognized regulatory point in the pigmentation pathway. Interestingly, sialylated oligosaccharides are functional in biological recognition systems involving cell-cell recognition and attachment [9]. Since melanosome transfer to keratinocytes is a rate-limiting step in skin pigmentation, this suggests that disruption of Neu5Ac(α2-6)Gal/GalNAc-oligosaccharide synthesis and/or function might inhibit melanosome transfer.

### Inhibition of EBL binding in melanocyte-keratinocyte co-cultures

There are more than 20 sialyltransferases in humans and sialyltransferase inhibitors have been reported [9].To determine the oligosaccharide sequence recognized by EBL, Shibuya *et al.* [9] reported a number of short oligosaccharides that showed inhibition of EBL-mediated precipitation of glycophorin, a highly sialylated glycoprotein. They deduced that the EBL lectin showed the highest affinity for oligosaccharides containing the Neu5Acα(2–6)Gal/GalNAc- sequence. We reasoned that this or similar structures might be useful targets for inhibiting melanocyte functions involving Neu5Acα(2–6)Gal/GalNAc-containing oligosaccharides. We thus tested for inhibitors that might affect melanocytes and keratinocytes in co-culture: 1) cytidine, a small molecule sialyltransferase (ST) inhibitor; 2) short sialylated oligosaccharides as competitive inhibitors of melanocyte-keratinocyte interactions, and 3) specific siRNAs vs the sialyltransfereases ST6 and ST3. None of these treatments affected cell survival (not shown). The results are detailed below.

### Effects of L-cytidine on EBL binding in melanocyte-keratinocyte co-cultures

**Fig. 3:**
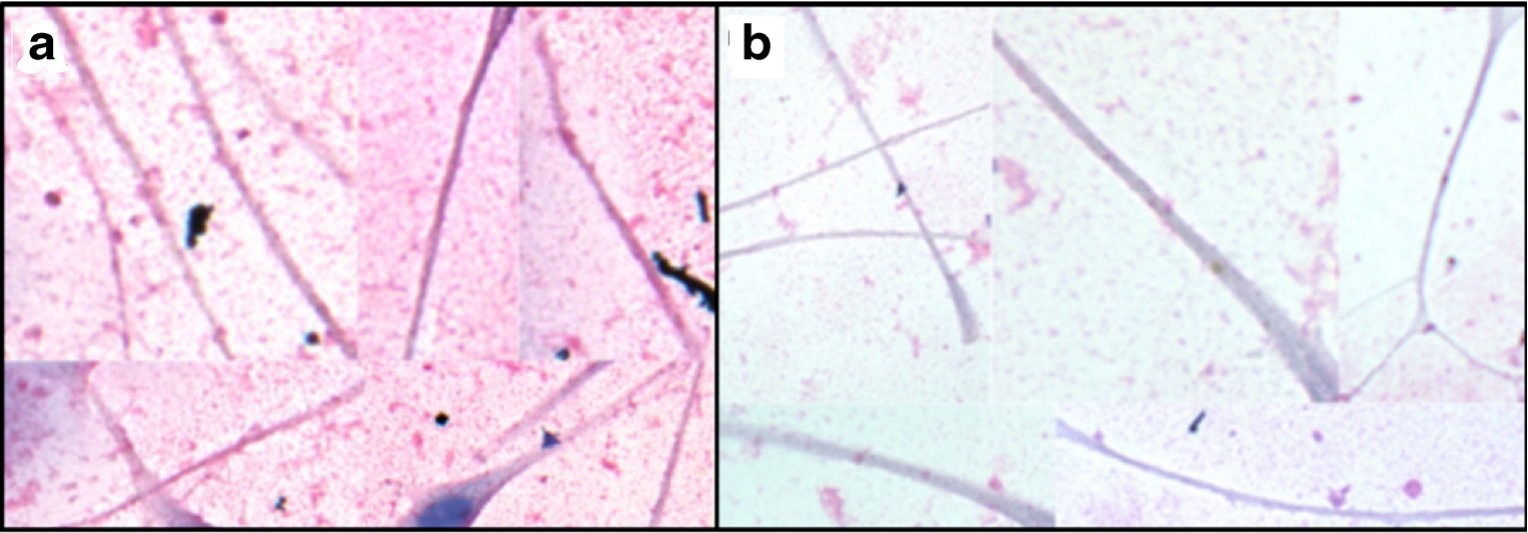
Effects of L-cytidine [25 μM] on EBL staining and melanin content of human melanocyte-keratinocye co-cultures. Cultures were incubated 72 h with L-cytidine (25 μM), rinsed in BSS, fixed with paraformaldehyde, rinsed again with BSS and stained with EBL (*red chromagen*) using standard IHC procedures. Counterstaining was with hematoxylin (*blue*). Random fields were photographed with a Zeiss Axioskop 40 light microscope equipped with a Spot Flex digital camera. Using Photoshop tools, dendrites were cut and pasted into the treatment groups herein. The composite images were then enhanced together with automatic contrast and brightening tools. **a**. Untreated controls; **b**. EBL stained

Experiments were carried out to test the effects of cytidine, an ST6Gal.I inhibitor (, on EBL staining in melanocyte-keratinocyte co-cultures (Fig. 3a and b). Untreated cultures showed prominent EBL staining of melanocyte dendrites, including filapodia in contact with keratinocytes (Fig. 3a). Treatment with cytidine markedly reduced EBL staining (Fig. 3b).

### Effects of short sialylated oligosaccharides as competitive apteninhibitors of EBL function

**Fig. 4:**
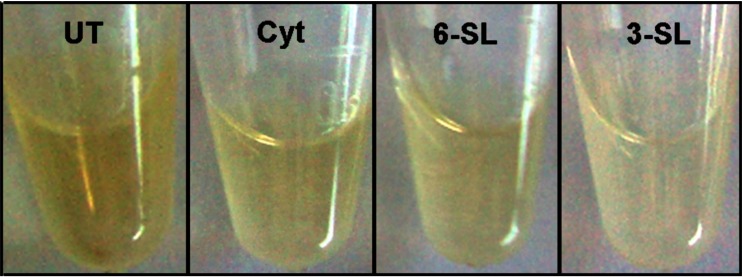
Effects of L-cytidine (Cyt, 50 μM), 6′-sialyllactose (6′-SL, 50 μM) and 3′-sialyllactose (3′-SL, 50 μM) on melanin content of human melanocyte-keratinocye co-cultures compared to an untreated control (UT). Cultures were incubated 72 h with each agent, pelleted by centrifugation, and melanin was solubilized for quantitation *via* spectrophotometry**.** Original images were produced with a Zeiss Axioskop 40 light microscope equipped with a Spot Flex digital camera. All images were enhanced together with automatic contrast and brightening tools

We next tested 6′-sialyllactose(Neu5Acα(2–6)Gal/GalNAcβ(1−4)Glc; 3′SL)Glc; 6′-SL) an oligosaccharide homologue of the EBL recognition sequence and a strong inhibitor of EBL-mediated precipitation of glycophorin [9, 10]. As a control, cultures were incubated with 3′-sialyllactose (Neu5Acα(2–3)Gal/GalNacβ(1−4)Glc; 3′SL)Glc; 3′SL) that does not interact with the EBL binding site due to steric hindrance, and consequently is a poor inhibitor of EBL-mediated precipitation of glycophorin [9, 10]. Cultures were incubated for 72 h with each agent, the cells were pelleted by centrifugation, and melanin was solubilized and quantitated through spectrophotometry. Unexpectedly, both oligosaccharides individually reduced melanin content compared to that in untreated cultures, and 3′-SL consistently gave the strongest reduction of the two (Fig. 4). This was surprising since 3′-SL is a poor competitor for EBL binding, as discussed above [9, 10]. Similarly, when melanocyte-keratinocyte co-cultures were stained for melanin with the Fontana-Masson silver staining procdure. 6′-SL and 3′-SL each reduced melanin content in melanocytes-keratinocyte co-cultures (not shown). Therefore, melanogenesis within melanocytes and transfer of melanosomes into keratinocytes were each inhibited by these agents.

### Effects of 6′-SL and 3′-SL in combination with cytidine on melanin content in human melanocyte-keratinocyte co-cultures

**Fig. 5:**
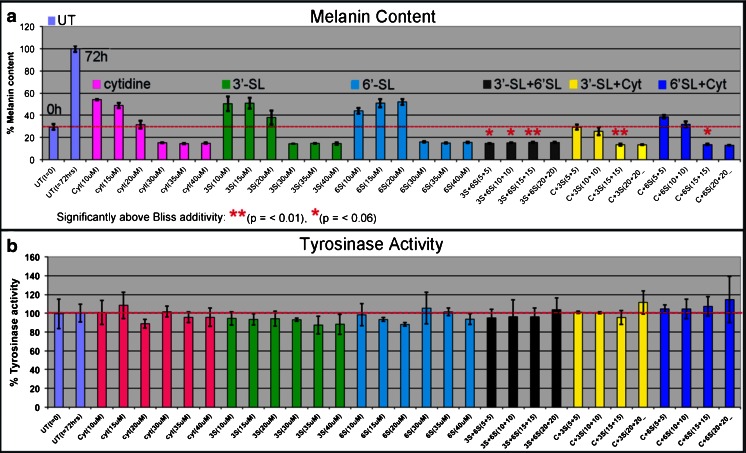
Melanin content (**a**) and tyrosinase activity (**b**) in melanocyte-keratinocyte co-cultures. The *red line* indicates the t_0_ levels at initiation of the experiment. Cases where melanin content was significantly greater than that expected for Bliss Additivity are indicated by (*) (*p* = ≤ 0.06); (**) (*p* = ≤ 0.01)

**Fig. 6:**
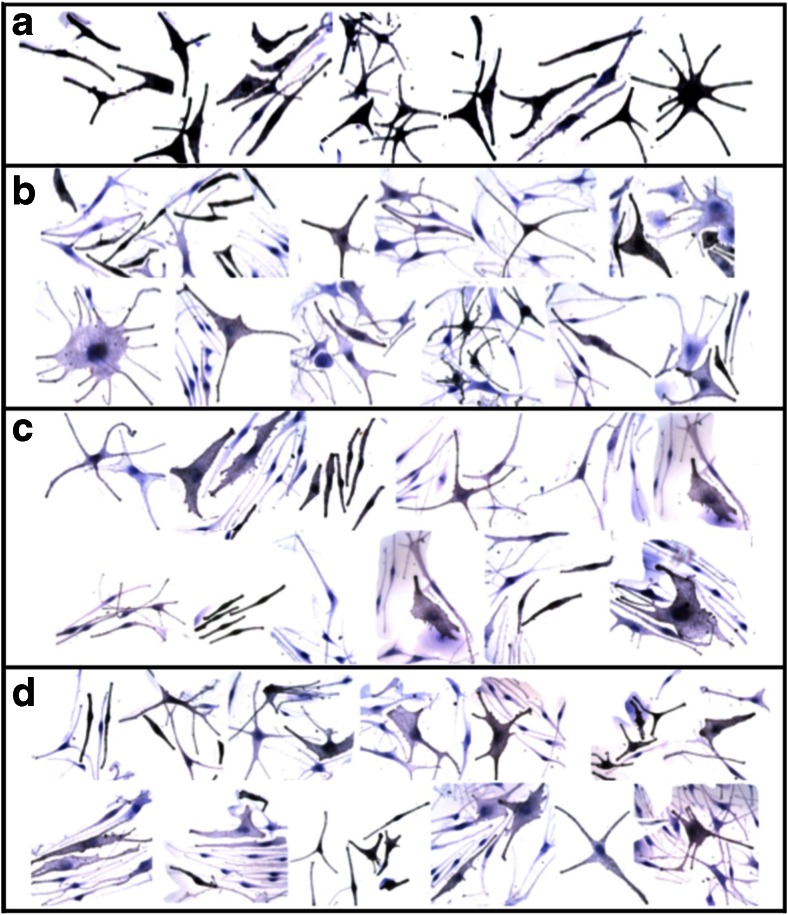
Effects of cytidine in combination with 3′-SL on melanocyte-keratinocyte interactions and melanosome transfer. Cells were incubated 72 h with cytidine + 3′-SL, fixed in paraformaldehyde, stained for melanin with the Fontana-Masson silver stain. Images were photographed and arranged into treatment groups with Photoshop tools. The images were enhanced together in the same layer with Photoshop automatic contrast and brightening tools. **a**. Untreated control. **b**. Cytidine (15 μM) + 3′-SL (15 μM) (**c**). 6′-SL (15 μM); (**d**). 3′-SL (15 μM). Magnification 63×

Dose response studies were carried out on melanocyte-keratinocyte co-cultures to compare the inhibitory activities of 6′-SL, 3′-SL and cytidine, as single agents and in combinations, on melanin content and tyrosinase activity. All categories caused highly significant reductions in melanin content at all concentrations tested (5–40 μM) (Fig. 6; Table 1). In some cases, treatments prevented new melanin synthesis during the 72 h experiment, *i.e.*, melanin above the t_0_ level at the beginning of the experiment (red line). In others, not only was new melanin prevented but, surprisingly, reduced below that seen at the t_0_ level during the 72 h experiment (Fig. 5b, red line), implying that these treatments caused melanin degradation and/or release to the culture medium. Cell viability was not affected by these treatments (not shown). The percent inhibition at each concentration was compared and analyzed for Bliss Additivity, *i.e.*, significantly the same as the sum of the two agents alone While many of the treatments indeed showed Bliss Additivity, others showed synergism, *i.e.*, inhibition significantly above that expected for Bliss Additivity. Synergism was seen in the combinations of 3′-SL + 6′-SL (5 + 5 μM, 10 + 10 μM, 15 + 15 μM); 3′-SL + cytidine (15 + 15 μM); and 6′-SL + cytidine (15 + 15 μM), with *p*-values ranging from *p* = ≤ 0.01 to *p* = ≤ 0.06 (Fig. 6a, red asterisks). In the same samples none of the agents significantly reduced tyrosinase activity (Fig. 6b). The results indicate that the inhibitors reduced melanin content by targeting post-tyrosinase pathways, possibly through interference with glycosylation processes.

**Table 1:** *P*-Value Tests: treated vs untreated control

Treatment	Conc. (μM)	*P*-value *vs.* untreated control
Cytidine	15	0.0003
	20	0.0001
	30	0.0004
	35	0.0005
	40	0.0004
3′-SL	10	0.0051
	15	0.0077
	20	0.0067
	30	0.0004
	35	0.0003
	40	0.0006
6′-SL	10	0.0002
	15	0.0013
	20	0.0027
	30	0.0006
	35	0.0003
	40	0.0006
Cyt + 3′-SL	5 + 5	0.0002
	10 + 10	0.0024
	15 + 15	0.0002
	20 + 20	0.0004
Cyt + 6′-SL	5 + 5	0.0007
	10 + 10	0.0020
	15 + 15	0.0005
	20 + 20	0.0003
3′-SL + 6′-SL	5 + 5	0.0003
	10 + 10	0.0005
	15 + 15	0.0002
	20 + 20	0.0005

*P*-values were determined by the Welch Two Sample *t*-test

The minimal doses for >80 % reduction of melanin content were determined for each treatment category. The combination of 6′-SL + 3′-SL was the most effective, causing ~85 % inhibition at the combined concentration of 5 μM + 5 μM, 3-fold more active than that in any other category (Fig. 6a, Table 2).

**Table 2:** Minimal treatment concentrations for >80 % reduction in melanin content

Treatment	Minimal concentration for >80 % inhibition
Untreated control	not applicable
Single Agents	
Cytidine	30 μM
6′-SL	30 μM
3′-SL	30 μM
Combined Agents	
Cytidine + 6′-SL	15 μm + 15 μM
Cytidine + 3′-SL	15 μm + 15 μΜ
6′-SL + 3′-SL	5 μm + 5 μM

Data are from Fig. 5a

### Effects of cytidine, 6′-SL, 3′-SL on melanin transfer

**Fig. 7:**
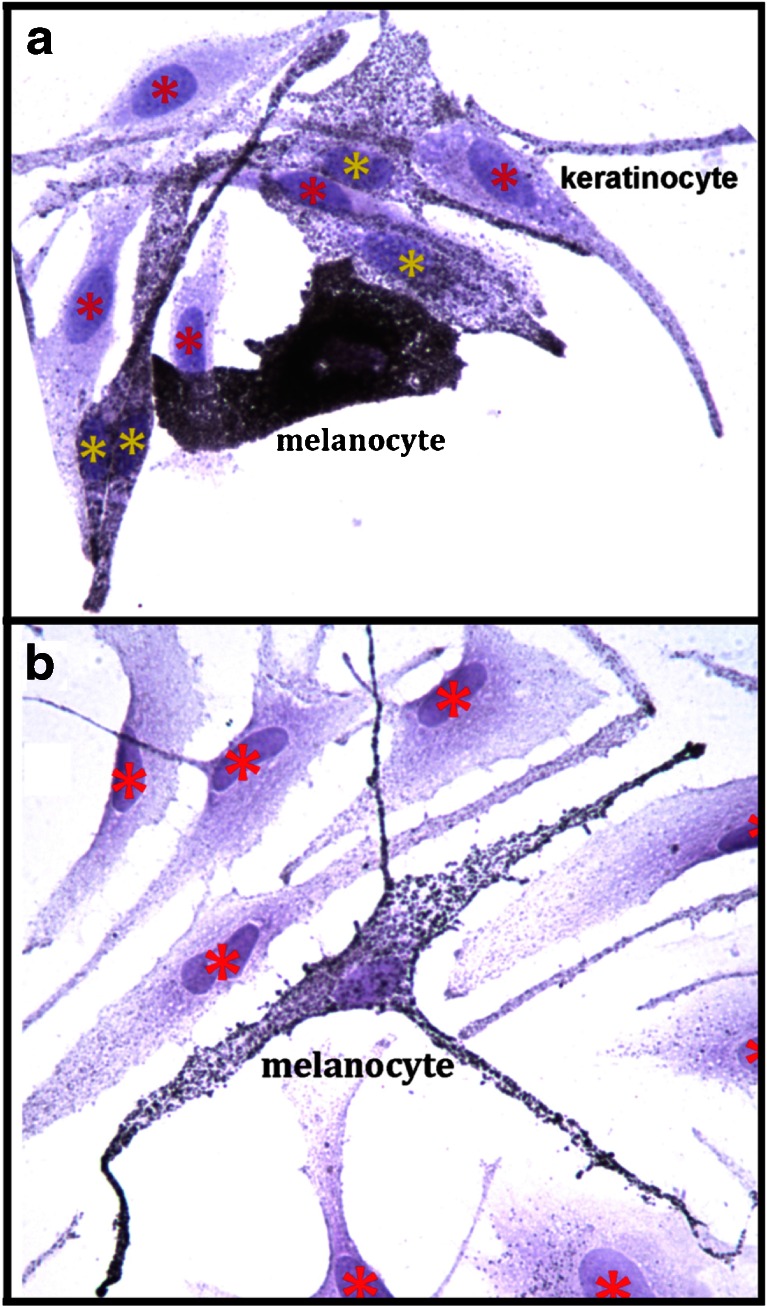
Effects of cytidine in combination with 3′-SL on melanocyte-keratinocyte interactions and melanosome transfer. Cells were incubated 72 h with cytidine + 3′-SL, fixed in paraformaldehyde, stained for melanin with the Fontana-Masson silver stain. Images were photographed at magnification 63× and arranged into treatment groups with Photoshop tools. The images were enhanced together in the same layer with Photoshop automatic contrast and brightening tools. **a**. Untreated control. **b**. Cytidine (15 μM) + 3′-SL (15 μM)

The effects on melanocyte-keratinocyte melanosome transfer were assessed after treatment with cytidine, 3′-SL, and 6′-SL alone and in combination (Fig. 7a and b). Untreated co-cultures had highly melanized melanocytes surrounded by keratinocytes. The melanocytes had close contacts with neighboring keratinocytes over large portions of the plasma membranes. The keratinocytes in direct contact with the melanocyte (nuclei with yellow asterisks) contained numerous cytoplasmic melanin granules transferred from the melanocyte. Keratinocytes not in contact with the melanocyte (nuclei with red asterisks) contained notably fewer melanosomes (Fig. 7a). In contrast a representative field from a co-culture treated with the combination of 3′-SL + cytidine showed a marked reduction in melanocyte-keratinocyte contacts and a reduction of melanosomes in both cell types (Fig. 7b). All treatment categories showed these effects, but since transfer is a dynamic process it could not be quantitated in the fixed cultures studied herein.

### Transfection of siRNAs for ST6 and ST3

**Fig. 8:**
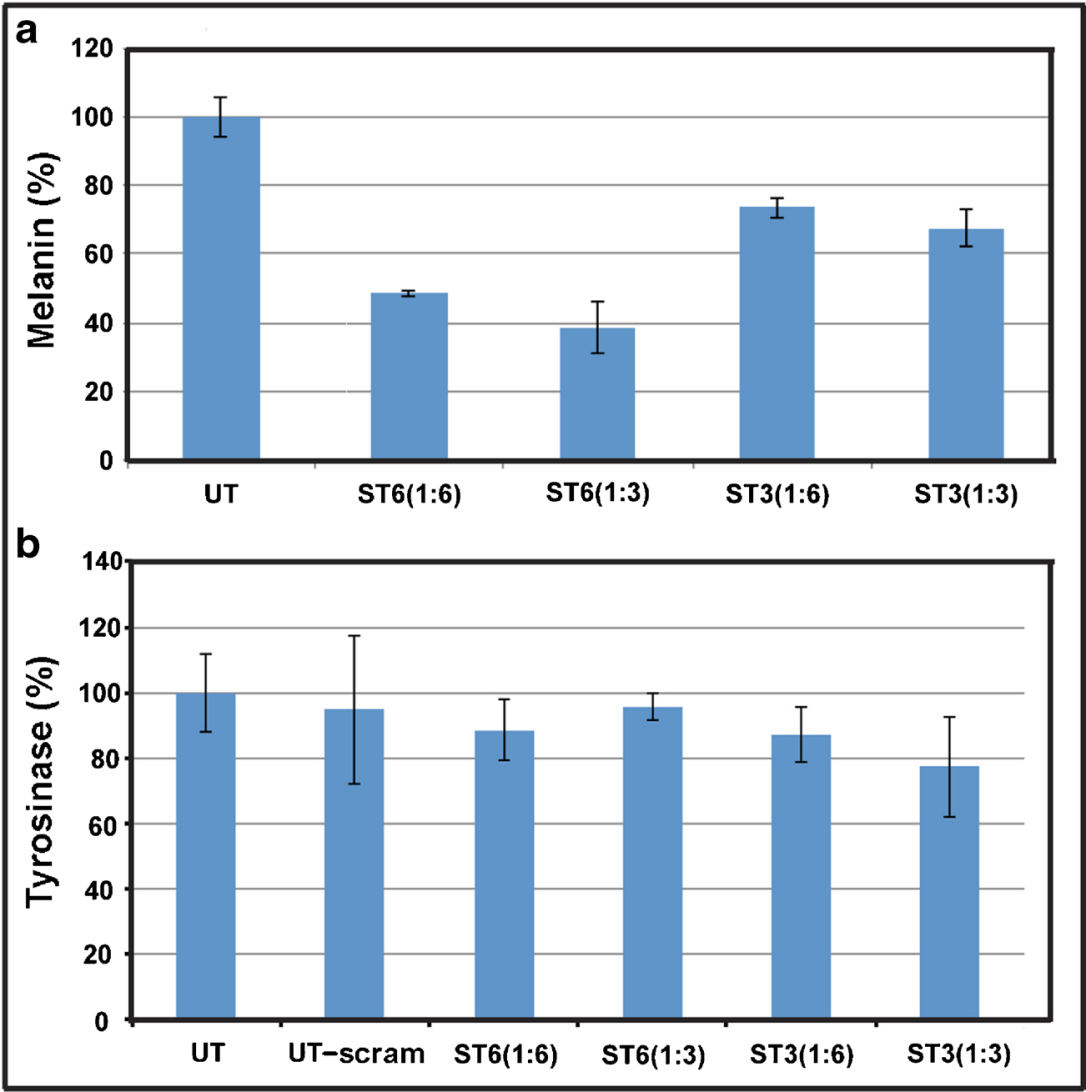
Effects of siRNAs for ST6 and ST3 on melanin content and tyrosinase activity in cultured human melanocytes. UT = untreated control; ST3 siRNA 1:6; ST3 siRNA 1:3; ST6 siRNA 1:6; ST6 siRNA 1:3

When ST6 and ST3 siRNAs were transfected into mouse melan-A cells, both EBL binding (Fig. 8a) and melanin content (Fig. 8b) were strongly inhibited by ST6 siRNA and to a lesser extent by ST3 siRNA.

## Discussion and conclusions

Together, these findings demonstrate for the first time that Neu5Acα(2-6)Gal- and possibly sialyl(α2-3)gal-terminated glycans play key roles in melanin synthesis and melanosome transfer to keratinocytes. The structures are prominently expressed on melanocyte dendrites where they are closely associated with the transfer of melanosomes to keratinocytes, a rate-limiting step in skin pigmentation. While it is likely that the glycans are glycoproteins, at this point we cannot rule out the possibilities of glycolipids or proteoglycans, nor do we know if the oligosaccharide is N- or O-linked in the glycoconjugate.

Excess or uneven skin pigmentation such as seen in melasma, post-inflammatory hyperpigmentation and solar lentigenes can cause severe anxiety and depression in affected individuals. These findings point to new possibilities for reducing hyperpigmentation through inhibition of the synthesis and function of oligosaccharides regulating the pigmentary system.

## Materials and methods

### Cell culture

Primary neonatal normal human keratinocytes (NHKs) (Invitrogen, Life Technologies, Grand Island, NY) were seeded at a density of 5 × 10^5^ cells/well into 4-well collagen coated chamber slides (Fisher Scientific, Waltham, MA). Cells were maintained in 1 ml of serum-free keratinocyte growth medium (KGM) (Keratinocyte-SFM, Invitrogen) and incubated for 48 h in 5 % CO2 incubator. Darkly pigmented human neonatal epidermal melanocytes (HEM-DP) (Invitrogen) were seeded over the keratinocytes at 2.5 × 10^5^ cells/well and incubated 24 h, 37^o^ C. The KGM was then replaced with 1 ml of melanocyte growth medium (MGM) (Invitrogen), and the co-cultures were incubated for another 48 h. The co-cultures were then treated with the inhibitors at indicated concentrations in a final volume of 20 μl/well in triplicate for 72 h. The cells were then gently rinsed with 1× phosphate buffered saline (PBS) and fixed in 4 % Para-formaldehyde (PFA) for 20′ at room temperature.

### Lectin histochemistry

Cells fixed in 4 % PFA were washed in 1× PBS and incubated for 10′ at RT in a dual enzyme block solution (Dako, Glostrop, Denmark) followed by wash in 1× PBS, and then incubated with protein block solution for 10′ at RT. The cells were then incubated with biotinylated elderberry bark lectin (1:800) (Vector Labs, Burlingame, CA) for 30 min at RT. Following a wash in 1× PBS, the cells were incubated with vectastain ABC-AP (Vector Labs) complex for 30 min at RT. The ABC-AP complex was prepared fresh according to the instructions provided with the vectastain ABC-AP kit. The cells were washed in 1× PBS to remove the ABC-AP complex and incubated with fast red chromagen staining solution (Dako) for 10′ at RT. The cells were rinsed in water and incubated with gill free hematoxylin for 5′. The slides were rinsed in H_2_O followed by a gentle wash with 0.5 % ammonium hydroxide. The slides were air dried and mounted on cover slips and the images were captured in tiff format using fluorescence microscope (Nikon) and analyzed.

### Fontana-Masson silver stain for melanin

Silver staining for melanin was performed according to the instructions provided with the Fontana-Masson staining kit (American MasterTech, Lodi, CA). Cells grown in collagen-coated chamber slides were fixed in 4 % PFA and rinsed with water for 5′ to remove traces of PBS. Slides were then placed in ammonical silver solution followed by incubation in solutions of 0.1 % gold chloride, 5 % sodium thiosulfate, rinsed in running tap water to remove the staining solution and dehydrated through 3 changes of fresh absolute alcohol. Cleared slides were rinsed through 3 changes of fresh xylene and coverslips were applied with mounting medium.

### Transfection of siRNA’s

Mouse melan-A cells and human darkly pigmented (DP) melanocytes were added at 8 × 10^4^ cells/well in a 24 well plate and incubated for 24 h at 37 ° C. They were then transfected with ST6 and ST3 siRNAs according to the RNAiFect Transfection Handbook (Qiagen). Briefly, 1 μg each of siRNA ST6 and siRNA ST3 was added to RNAi Fect transfection reagent at dilutions of 1:3 and 1:6, complexed with 3 μl and 6 μl of the RNAiFect in 100 μl of culture medium, incubated for 10–15 min with Melan-A and DP cells in triplicate wells at room temperature and then an additional 24 h at 37 ° C. The cultures were rinsed, fresh culture medium was added and the cells were incubated an additional 24 h at 37 °C. The experiment was stopped by discarding the culture medium and washing with 1× PBS three times. The cells were then treated with lysis buffer and melanin was isolated by alkaline hydrolysis and quantified by absorbance at 490 nM.

### Treatment with inhibitors

Cells were plated on 24-well plates at 2 × 10^5^ cells/well and treated with various inhibitors in triplicate. Media were replaced with fresh inhibitor-containing media every 24 h. After 3 days, cells were lysed with cell lysis buffer containing protease inhibitor cocktail and PMSF (phenylmethyl sulfonyl fluoride) (Invitrogen) and incubated 20’ min on ice. The lysed cells were centrifuged at 10,000 rpm for 10′. The supernatants were saved for measurement of dopa oxidase activity and the pellets were evaluated for melanin content.

### Dopa oxidase assay

Reaction mixture contained 20 μL of the lysate supernatant, 20 μl of 10 mM L-Dopa in 160 μl of 50 mM sodium phosphate buffer (0.1 M, pH6.8) Samples were transferred in triplicate to 96-well plates and evaluated for the formation of dopachrome by reading the absorbance at 475 nm for 30′ at 1′ intervals on an M5 microplate reader (ThermoScientific, Waltham, MA). The slope derived from the kinetics was used to calculate the % dopa oxidase/tyrosinase activity in treated wells relative to the untreated controls.

### Melanin assay

Melanin was extracted following the procedure of Ni-Komatsu, *et al.*, (2005). In brief, the growth medium was removed and the cells lysed as above. The lysed cells were centrifuged and the pellet was washed with ethanol:ether (1:1) solution and then solubilized in 100 μl 20 % DMSO in 2 N NaOH. The melanin extracts (100 μL) were transferred to a 96-well plate and total melanin content was quantitated with a M5 Spectramax plate reader at 490 nm.

### Photography and image processing

Representative fields of cultured cells were photographed with a Zeiss Axioskop 40 light microscope equipped with a Spot Flex digital camera. Using Photoshop tools, areas of interest were cut and pasted into the treatment groups herein. For a given figure, any composite images were enhanced together, in a single layer, with automatic contrast and brightening tools.

### Statistics

Dose response curves were assessed for Bliss Additivity and synergism. For comparisons of melanin content and tyrosinase/dopa oxidase activity in treated *vs.* untreated cultures, *P*-values were determined by the Welch Two Sample *t*-test.
